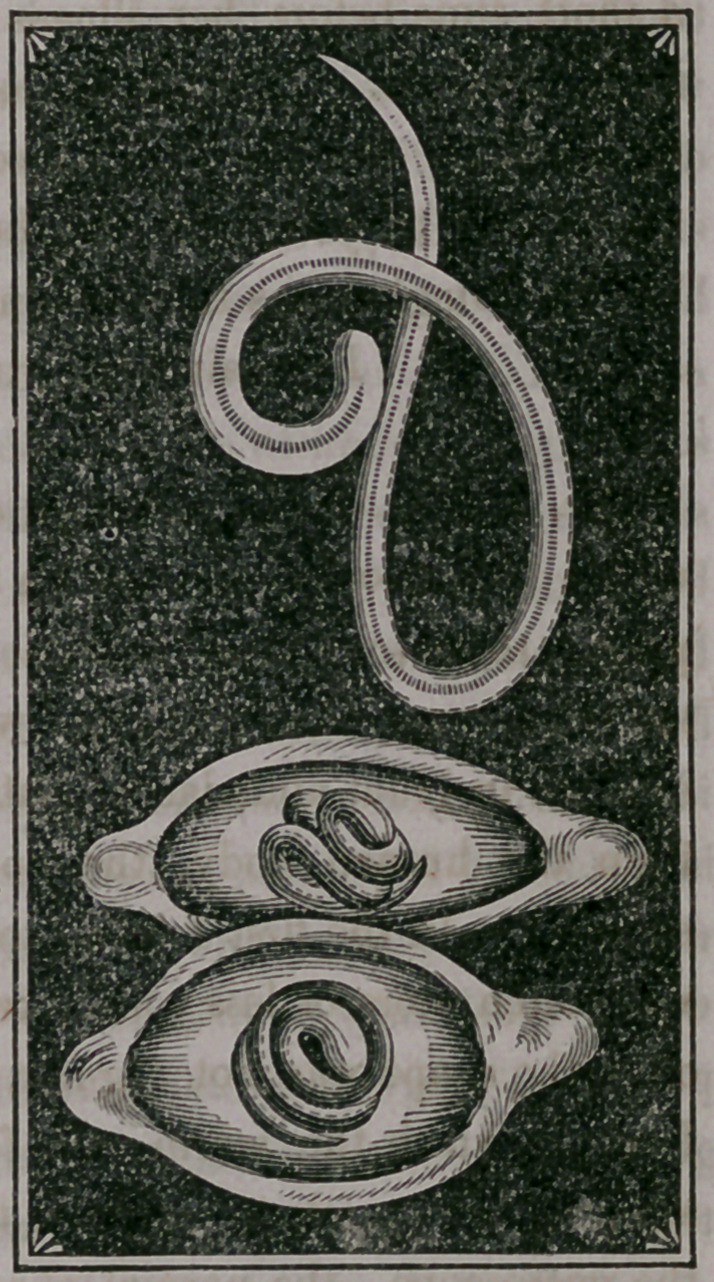# The Newly Discovered Disease Depending upon Trichina Spiralis, in Erie County

**Published:** 1864-06

**Authors:** 


### THE NEWLY DISCOVERED DISEASE DEPENDING UPON TRICHINA SPIRALIS, IN ERIE COUNTY.

In our last number we made reprint of an account of this disease, its occurrence in Germany and the fatality attending it. Since then we have been startled by the discovery of this same disease in a neighboring town. Dr. Krombein of our city in connection with Dr. Dingler of Lancaster, has discovered the cause of death in several instances to be *trichina spiralis*, which is found in immense numbers in the muscles of the victims. Dr. Krombein has sent us specimens of the muscular tissue obtained upon *post mortem* examination, which are seen on microscopic examination to contain innumerable trichinæ. We have also received portions of the sausage and ham, of which the unfortunate victims had eaten, and these also contain immense numbers of these parasites. Dr. J. R. Lothrop and Prof. George Hadley have also made careful examination of these specimens, and Dr. Lothrop's report will be seen in connection with the interesting communication from Dr. Krombien. These cases of trichinous disease are the first in this country positively traced to this cause, though it is stated in the *American Medical Times*, February 20, 1864, "that during the past week an instance of the poisoning of a whole family, and the death of one member, caused by eating a ham, occurred in the city of New York. The case was investigated by Dr. Schnetter, who found the ham full of trichina spiralis, and did not hesitate to attribute the poisoning to this parasite."

It will be seen that in the cases reported by Dr. Krombein the history is complete—he has not only found the worms in the ham and sausage, but also in the muscles of the patients who have died, leaving no doubt as to the cause of death. This is important, since nothing less than perfect demonstration can be satisfactory in the investigation of a disease, which has been denominated *new.* "Three disgusting and dangerous diseases in man thus owe their origin to the ingestion of the flesh of the pig, viz: tape-worm, hydatids and trichina."

*Dear Doctor:*—At your request I give you herewith the history of the two cases of Trichiniasis that occurred lately in Cheektowaga. I was invited by my friend Dr. Dingler of Lancaster, on Sunday, the 15th of May, to see with him two patients whom he had attended for four days.—T. F——, a blacksmith, aged 30 years, and his wife aged 20 years, were taken ill simultaneously the 29th of April with stiffness of the limbs and the whole body, bloating of the face, with a slight œdema of the eyelids; soon after there followed distinct pains in all the limbs and body, so that they could not bear even the slightest touch. By and by the pains diminished; there set in very labored respiration and great prostration, combined with profuse sweats. In the commencement of the illness they both had had, slight diarrhœa for a few days, and during the whole course of the sickness they suffered greatly from sleeplessness and unquenchable thirst. The woman, who was in the 3d month of pregnancy had aborted the 12th, and from that time there was œdematous swelling of both lower extremities. Fever in both patients was very high, (pulse 138 in the man, 146 in the woman,) but the skin was not hot, but rather cool. I considered at first, like Dr. Dingler, both cases to be "acute muscular rheumatism," of a somewhat peculiar character, but during my return home I suddenly remembered to have read some months ago in a German Medical Journal (*Medicinische Chirurgische Monatshefte,* September number, 1863,) of some cases of Trichiniasis which resembled very much the two cases above stated, and therefore I immediately wrote to Dr. Dingler that as soon as one of the patients should die, he might send me some particles of muscle for microscopical examination. Two days afterwards he visited me, telling that the man had died yesterday evening, (the 16th of May, at 9 o'clock P. M.) We went there and found the woman dead also, (she died the 17th at 11 o'clock A. M.,) and cut from both some particles of the muscles of the thorax, the abdomen and the thigh. The microscopical examination in which Dr. Homburger kindly assisted me, disclosed many trichinæ, both in the encysted and in the free state.

I wish to say further, that at the time Dr. Dingler and myself saw the said patients, he told me that he had another family under treatment, having the same disease, residing about two miles distant, from the above patients, (at Marilla,) the wife was a daughter of this same family, who a short time before her illness paid a visit in company with her husband to her parents. This family consisted of seven members—the man about 60 years of age, the wife 55 years, and five children respectively 24, 22, 18, 14 and 12 years old. Father and mother are dead, children still alive but Dr. Dingler says they are in a dangerous situation.

I remain very truly yours,

L. KROMBEIN, M. D.

*Mr. Editor:*—The specimen of human muscle taken from a person after death, and also the sausage he had eaten of, which you brought me, supposing them to contain the Trichina Spiralis, I carefully examined under, the microscope, both alone and with Dr. Hadley. The parasite was found in both, in great abundance, but in different states. In the muscle taken from the human body, the worm was free, while in the sausage it was encysted. In the first I failed to find the worms enclosed in a cyst. They were often more or less coiled, two or three turns of an eliptical form; but often the shape was not regular, though seldom straight. The irregular forms in which the worm was found were probably caused by tearing or scraping the muscle to render it thin enough to become transparent under the microscope—the normal shape being more or less coiled. Under the microscope the worm could be readily seen with a low power, and presented uniformly a pointed head, a body increasing in size to the tail, which had somewhat of a truncated appearance, with a slight fissure. There was an appearance of an intestinal canal running the whole length of the body, somewhat undulating, and filled with granular matter. The appearance is here represented magnified 100 diameters. With a high power, the external covering of the body appeared made up of rings, somewhat after the articulate plan of structure, which gave fine markings across the body, and on the inner or concave side of the curves, a serrated appearance. In the neighborhood of the worms there were often numerous fat globules, and in some portions of the muscle, an abundance of granular matter. The elementary fibres of the muscle were in many places distinct, but in others less so, there being appearances as if the sheath or sarcolemma of the fibres had been emptied of its contents and collapsed. The worm could be easily seen through the muscular fibres, and was detected often lying transversely to their course.

In the portions of the sausage examined, the worms were enclosed in an ovoid cyst, and here were found free. Even scraping the muscle did not rupture the cyst. They were much smaller than the free worm, were always coiled, occupying the centres of the cyst, and in most cases single. One cyst only was observed in which were two worms, separate from each other, each occupying an extremity of the cyst. The cysts were enclosed by the muscular fibres, which had the appearance of having been pushed aside, and at either end, the space where the muscular fibres separated was filled with fat globules. I did not find a cyst without a worm. The appearance was as here represented, magnified over 200 diameters. The worm occupied about one-third of the cyst space. In one small piece of the muscular tissue, of the sausage, I should rather say small collection of scrapings, nearly thirty cysts containing worms were counted. I made no attempt to ascertain the nature of the other cyst contents. It appeared to be filled with transparent fluid, which did not extend into its prolongations.

The worm appears then, in this case in the encysted state in the sausage, and in the free state in the human muscle. It would therefore appear probable, that taken into the stomach in the encysted form, the worm escaped from its envelop by being set free in the process of digestion, and immediately set out on its progress towards its ultimate destination, viz: the striated muscles. The first step was, probably, piercing the intestinal walls, thereby creating the iniatory diarrhœa; afterwards passing through the peritoneum on its migration outwards. Arriving at the muscles, its abode is fixed in them. Its ultimate destination is a matter of doubt.—The opinions of observers will be given on this subject presently. The observations in this case only extend to the two states in which the worm was found, it being a conjecture merely, that the encysted state is an immature or embryotic one. It is also merely conjecture, that removal from the animal in which it exists in this state of cyst-life, to another, is an essential step in the perfection of its development, as in the case with the cysticercus cellulosæ which passes its cyst-life in the tissues of one animal, and develops the fully formed tape-worm in the intestines of another.

Perhaps some process of reproduction would be necessary to account for the immense number of worms which probably existed in the body of this trichinous patient. For there is reason to suppose, that all the muscles as well as the piece of the pectoral muscle examined, were infected. It is difficult to believe that as many worms could have been taken into the stomach in the portion of sausage which one person would eat, as must have existed in the body of the patient, to have proved fatal by their presence. Yet it may have been so. For when we consider that a minute scraping was found to contain about thirty worms, the number in a piece of sausage as large as would be ordinarily eaten by a healthy man, must have been immense. If such process of reproduction took place it must have been rapid, since death followed close upon the eating. The fact that the free worms were of different sizes, may give a show of probability to the idea. But then again, these encysted worms being asexual, and as it were in a state of transition, are supposed to be incapable of producing in any manner a progeny similar to themselves. This may not be true of the free worm, which may be in a higher state of development, and therefore may produce a progeny similar to itself even in a short time. This case may give rise to some general considerations respecting the mode of life, etc. of this worm. Its seat is well known to be the striated muscles, with the exception of the heart and the sphincter ani externus, in which it has not hitherto been found. Its diffusion is sometimes so extensive, that the smallest muscles, those of the eye, tympanum and larynx, do not escape.

Several migrations seem necessary to its perfect development. If the ova are swallowed, either before or after the development of the embryo, they may arrive at the tissues by two avenues, viz: by the blood, or by penetrating the intestinal walls. In the latter case, a solution of the enclosing capules which contain the embryo must take place. When capsules enclosing worms, in an immature or embryonic state, visible under the microscope, are taken into the stomach, it does not seem doubtful by what way they would arrive at their destination, viz: by boring through the intestinal and other tissues.

The cyst is not of uniform shape, being generally oval, though it may be nearly circular, or on the other hand elongated at the extremities. The cyst may be opaque or transparent, according to the length of their residence in the muscles. Sometimes the cyst walls have depositions of live salts, more abundant as the age increases. Some maintain that the worm easily escapes before some calcareous incrustation takes place. It has been a question whether the cyst was formed by the re-action of the tissues, or was a part of the worm structure, being in fact the result of a kind of metamorphosis of the animal. This view is supported by the regular form of the cyst. Authorities, however, adopt both methods of formation, and the migration must be followed by exudation which would furnish the material for the capsule, if the formative power would tend to such a shape. Usually there is but a single worm in a cyst, but it may, in instances, contain two or more. The worm is tenacious of life, and endures unhurt considerable degrees of heat and cold. It may not be destroyed by moderate frying, and it survives the process of smoking, to which preserved meats are subjected. In the cyst it is motionless, but observers state that movements have been seen when free. The cyst fluid is transparent, and generally occupies a circular or oval space in it, not extending to the prolongations,

The trichinæ, like all encysted entozoa, are by most observers, considered as the imperfectly developed young of other parasites. They are therefore in a kind of embryonic condition. Reasoning from what is known of other encysted worms—of the cysticerus cellulosæ for instance—we should suppose them incapable of higher development in the situation in which they are found. They must migrate or become abortive and die. But nothing is satisfactorily known, and no other migration has been ascertained. What becomes of those that escape from their cysts is unknown. They are stated to remain in the human body encysted, thirty or forty years. Küchenmeister states that there is no doubt that those found in the muscles of man mostly die, becoming finally calcified in their cysts. No experiments or observations have ever established the fact of a higher development. The same authority, however, states his belief that the trichinæ are the young, undeveloped brood of the trichocephalus dispar, a long thread worm, which is found in the human cæcum. This worm has separate sexes, and is therefore capable of producing its like, though it must undergo several migrations and transformations before complete development. Those who wish to know his ground for this belief, will do well to consult his work on parasites, from which the above general statements are derived.

It should be further stated, that Herbert maintains a triple mode of life for the trichinæ, namely, in the encysted state; quite free, which he regards as living free, being developed from eggs borne by the blood; and lastly half-free, in cases of the peritoneum, and much larger than the first and second forms. He believed that free worms had no power to encyst themselves. He also gave trichinous flesh to dogs and pigeons, and they all became trichinous. No experiments by others have had like results.

It remains, only, to add that no especial pathological importance has heretofore been attributed to the presence of the trichina spiralis in man. It has appeared that the immigration of the trichina caused no special reaction, and that its residence for many years was borne without injury. The cases related by Dr. Krombein appear to establish the fact, that it may prove fatal.

If the observation proves correct that all worms existing in the human muscle examined, were free, their final destination, had their presence not proved fatal, would be of much interest. Would they after a time become encysted, and after a long time die and become calcified? Or in progressing in their development arrive at a higher state, and thereby infect the body with the trichocephalus dispar? Purely zoological reasons would determine to the belief that such destination was probable, inasmuch as the encysted worms are but imperfectly developed young of other parasites, and that a migration is necessary to complete transformation. But while facts in the developmental history of this worm are wanting, no positive opinion is possible. It is beyond the power of any one to state, how they can reach situations in which the higher development can take place.

J. R. Lothrop.

## Figures and Tables

**Figure f1:**