# Errata

**Published:** 1864-07

**Authors:** 


ERRATA.—In the article communicated by Dr. Lothrop on Trichina, in last number, page 433, 2d line, for *here* read *none.*
" 434, last line, for *live* read *lime,*
" 434, 2d line from bottom, for *cyst* read *cysts.*
" 435, 2d line, for *easily* read *rarely.*
" 435, 9th line from bottom, for *a* read *or.*
" 435, 3d line from bottom, for *Herbert* read *Herbst.*
" 436, 1st line, for *cases* read *sacs.*

There are several other errors caused by omission of a letter, as *capules* for *capsules*, and *cysticerus* for *cysticercus*, but as the meaning is apparent, they need not all be pointed out. The last revise of the proof-shoots was by mistake of printer overlooked.

